# Bibliometric Analysis of Global Research on Clavulanic Acid

**DOI:** 10.3390/antibiotics7040102

**Published:** 2018-11-26

**Authors:** Howard Ramirez-Malule

**Affiliations:** Escuela de Ingeniería Química, Universidad del Valle, A.A. 25360 Cali, Colombia; howard.ramirez@correounivalle.edu.co; Tel.: +57-2-321-2100 (ext. 7367)

**Keywords:** clavulanic acid, *Streptomyces clavuligerus*, bibliometric analysis, resistant bacteria, antibiotics, multidrug resistant bacteria

## Abstract

Clavulanic acid (CA), a potent inhibitor of the β-lactam, ase enzyme, is frequently co-formulated with a broad spectrum of antibiotics to treat infections caused by β-lactamase-producing pathogens. In order to evaluate the impact and the progress of CA studies in the last four decades, a bibliometric analysis of the global scientific production of CA was carried out. A total of 39,758 records in the field of CA were indexed in the Scopus database for a 43-year period of study (1975–2017). The results indicated that CA studies have grown, showing three phases (1975–1999, 2000–2003 and 2004–2017) based on records of publications; the results showed a sigmoidal profile. Medicine was the main subject area for CA studies, whereas biochemistry, genetics and molecular biology were areas of research for CA production by *Streptomyces clavuligerus* (*S. clavuligerus*). Nevertheless, chemical engineering (as a subject area) had the highest increase in the percentage of publications related to CA production by *S. clavuligerus*. The United States, France, the United Kingdom, Spain and Brazil were the leading countries in the scientific production of studies on both CA and CA related to *S. clavuligerus*. This analysis allowed the identification of the area of knowledge with the highest impact on CA studies, the top researchers and their geographic distribution, and also helped to highlight the existence of antibiotic-resistant bacteria as an emergent area in CA research.

## 1. Introduction

Many microorganisms have the ability to produce bioactive compounds (or compounds to be further modified for higher efficacy) that can serve as an effective drug(s) for treatments against multidrug-resistant bacteria [1,2,3,4,5]. Within the bacterial domain, actinomycetes have been reported as producers of anticarcinogen, antioxidants, antivirals and antibacterials [4,5,6,7,8]. In this regard, *Streptomyces clavuligerus* (*S. clavuligerus*), which belongs to the *Actinobacteria* family, is an aerobic and Gram-positive bacterium that produces β-lactam antibiotics, such as cephamycin C and clavulanic acid (CA) [9]. CA is a potent inhibitor of the β-lactamase enzyme, and is currently co-formulated with a broad spectrum of antibiotics, leading to global health benefits [10,11,12]. Studies on bibliometric analysis have been used by researchers to examine and assess the specific growth of publications on medical research [13,14,15,16]. This type of analysis helps to detect weak and strong areas in this field that eventually suggest the creation or modification of government policies. Despite the hundreds of CA studies published, a bibliometric analysis of the global scientific production is still missing. Since CA has been playing an important role in global health, it would be beneficial to provide a comprehensive view and analysis of the status of the research in this field.

In this study, an analysis of scientists performing CA research along with CA studies by *S. clavuligerus* and its connection with chemical engineering is presented. This analysis allowed the identification of the area of knowledge with the highest impact on CA studies, the top researchers and their geographic distribution, and helped to highlight the existence of antibiotic-resistant bacteria as an emergent area in CA research. All of this information provides insights to create a multidisciplinary research strategy to develop new collaboration and funding opportunities in the CA field.

## 2. Results and Discussion

### 2.1. Quantification of Clavulanic Acid Studies

**Figure 1:**
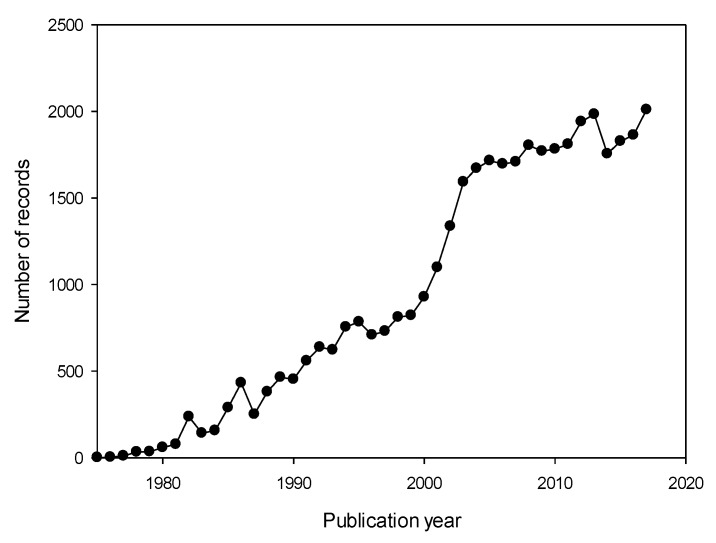
Quantitative growth process of scientific studies concerning clavulanic acid in the period of 1975–2017.

Guided by the approach presented in the methods, 39,758 documents were found in the Scopus database in the period of 1975–2017. Figure 1 shows the profile of the number of publications per year. The results can be divided into three periods based on the growth of the number of publications: (i) 1975–1999, (ii) 2000–2003 and (iii) 2004–2017 (Figure 1). In total, 9460, 4956 and 25,342 documents were indexed for the above-mentioned periods with 378, 1239 and 1810 documents per year on average, respectively. Nonetheless, the maximum growth of publications—223 documents per year (see the slope in Figure 1)—was observed in the second period.

The research (in the entire period of study) was related to the following areas:

- Medicine.
- Pharmacology, Toxicology, and Pharmaceutics.
- Immunology and Microbiology.
- Biochemistry, Genetics, Molecular Biology.
- Veterinary.

**Figure 2:**
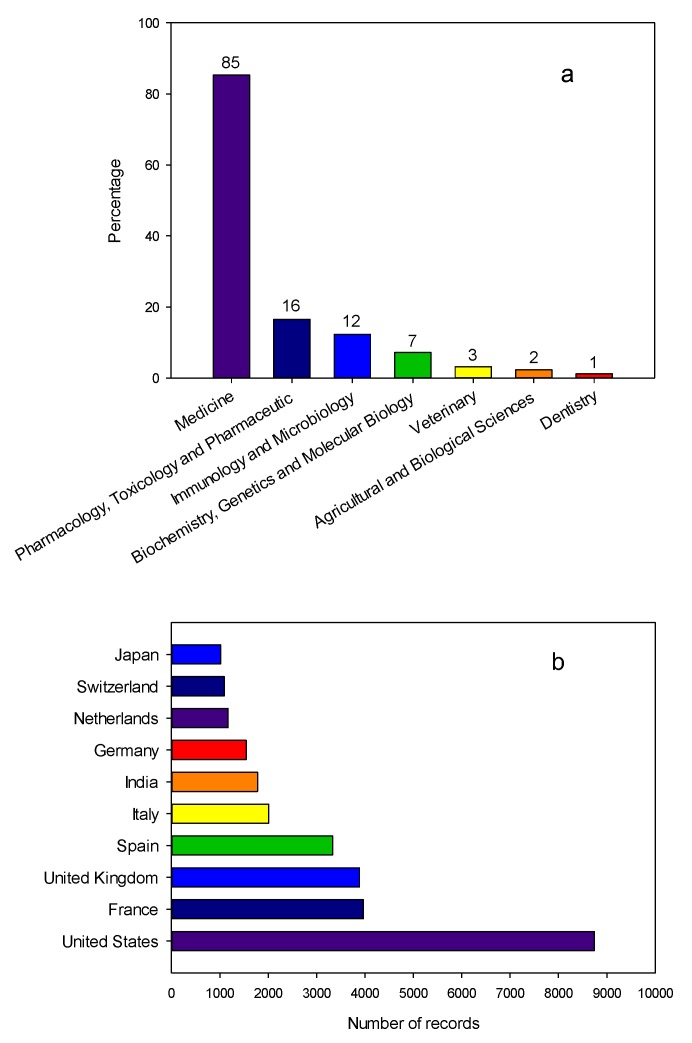
Summary of publications on clavulanic acid between 1975 and 2017. (**a**) Documents grouped by areas. Note: percentages add up to more than 100 because a document can be indexed in more than one area. Other areas contributed 6.2%. (**b**) Top 10 leading countries in the field of clavulanic acid.

It is to be expected that medicine occupies first place in the research reports since CA is co-formulated with a broad spectrum of antibiotics to treat infections caused by β-lactamase-producing pathogens (Figure 2a) [9,11]. The top 10 researchers (according to number of publications) in the field of CA are presented in the Supplementary Materials (see Table S1). Likewise, the top 10 leading countries concerning CA studies are presented in Figure 2b, where the United States occupies first place with 8741 documents, and France occupies second place achieving 3966 documents. The United Kingdom had a similar record to France (3886 documents). It should be noted that the main pharmaceutical companies are located in the United States, France and the United Kingdom, e.g., Pfizer, Sanofi, among others. As an example, GlaxoSmithKline (GSK) obtained sales of £165 million in the first quarter of 2018 for a drug marketed as Augmentin^®^ (a combination of amoxicillin and CA) [17].

#### 2.1.1. Sigmoidal Profile of CA Academic Studies

Based on the bibliometric analysis, it is possible to hypothesize why the number of publications became stable recently or why it had an exponential phase between 2000 and 2003. In fact, the bibliometric analysis served as an indicator of the progress of this field and made it possible to detect the changes in the research trend. Additionally, the evolution of the number of documents was combined with the search and analysis of key events that boosted the investigation of this field. Thus, after an analysis of the history of antibiotics—in particular of the records of the unique β-lactamase enzyme identified until 2010 [18] which was an indicator of growing bacterial resistance—one can observe that these records had similar profiles (sigmoidal) in terms of the number of publications about CA (Figure 1) (see also Supplementary Materials, Figure S1). Additionally, a keywords analysis of CA studies in 1987, 2002 and 2010 (a selected year within 1975–1999, 2000–2003 and 2004–2017, respectively) was conducted (see Supplementary Materials, Tables S2–S4). Keywords related to bacterial resistance appeared in the second and third period, which was in agreement with the increase in bacterial resistance in those periods. In addition, *Streptococcus pneumoniae* and *Staphylococcus aureus* also appeared since they are the most common cause of bacterial pneumonia.

#### 2.1.2. Genomic-Based Methods and Genome Sequencing

The second period in Figure 1 (between 2000 and 2003) was, among others, due to advances in genomic-based methods which triggered a wealth of available bacterial genome sequencing data, i.e., information related to drug discovery [19]. This information along with other disciplines, such as molecular genetics, molecular biology, biochemistry and bioinformatics contributed to the increase in CA publications (see also Supplementary Materials, Table S3) [18,19,20].

#### 2.1.3. How to Increase the CA Academic Studies?

**Figure 3:**
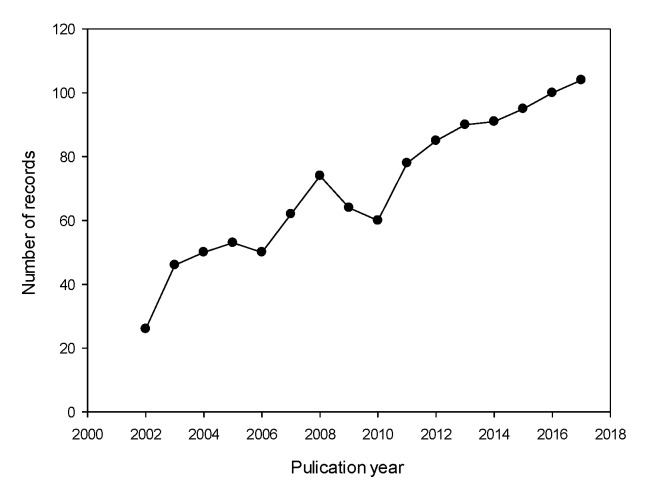
Records of amoxicillin-clavulanic acid (CA) resistance studies between 2002 and 2017.

Since the antibiotics market is considered a dynamic sector, one can expect that its academic studies obey a similar behavior. For example, an increment of antibiotic-resistant bacteria has been observed in the last 20 years (see also Supplementary Materials, Tables S3 and S4) [21,22]. Therefore, further studies in the previous subject area are required to overcome this world health problem. In this situation, antibiotic-resistant bacteria could be an area to focus on in the coming years; this would increase CA studies on productivity and/or related topics such as new and effective antibiotic treatments. In this regard, the number of published documents related to amoxicillin-CA resistance increased 4-fold from 2002 to 2017 (Figure 3) (see also Supplementary Materials, Figure S2).

#### 2.1.4. Side Effects of CA

Another alternative for CA studies is antibiotic side effects. For instance, despite the effectiveness of amoxicillin/CA in the treatment against pathogenic bacteria with resistance to β-lactam antibiotics, it is also considered one of the most frequently associated drugs with drug injury; however, further studies are required to analyze drug integration between amoxicillin/CA and concomitant potential hepatotoxic drugs [23,24,25]. Additionally, some drug eruption to CA has been reported, e.g., a patient developed a drug eruption on the day following amoxicillin and CA administration, which was considered a delayed hypersensitivity reaction [26]. These negative effects on human health should eventually be considered for future research.

### 2.2. Clavulanate Studies

**Figure 4:**
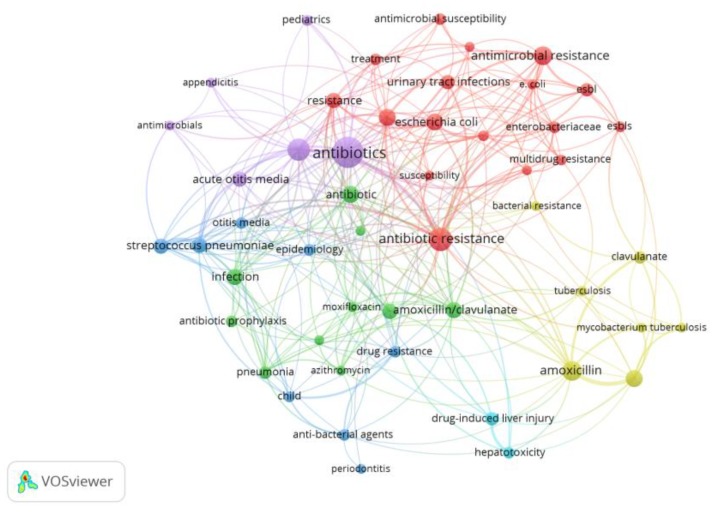
Research topic map for publications related to clavulanic acid and clavulanate studies.

As CA is marketed as a combination of amoxicillin and potassium clavulanate, this study aimed to explore the effect of adding clavulanate to the CA search. In this regard, 4343 documents were indexed between 1977 and 2017, 1574 of which were published in the last 10 years. Figure 4 shows the research topic network visualization of publications related to CA and clavulanate in the period between 2008 and 2017 (six clusters were observed which are represented by different colors). Again, antibiotic resistance appeared as a key node (the second biggest node in the network), confirming the preponderant role of bacterial resistance in CA studies (Figure 4). The authors’ keywords, antibiotic resistance, antimicrobial resistance, resistance, drug resistance and multidrug resistance, had 56, 38, 25, 14 and 13 occurrences, respectively. The top 10 author keywords with their number of occurrences are shown in Table 1.

### 2.3. Antibiotic and Multidrug Resistance Studies

To this point, bibliometric analysis conducted in this study has shown that bacterial resistance plays an important role in CA studies. Therefore, this study aimed to explore the effect of adding “antibiotic resistance” or “multidrug resistance” to the CA search.

#### 2.3.1. Antibiotic Resistance

In total, 1943 records were indexed between 2015 and 2017. The top 10 author keywords with their occurrences are shown in Table 2. It draws attention to the “drug resistance” and “multidrug resistance” keywords that appear in the last two positions of Table 2.

#### 2.3.2. Multidrug Resistance

**Figure 5:**
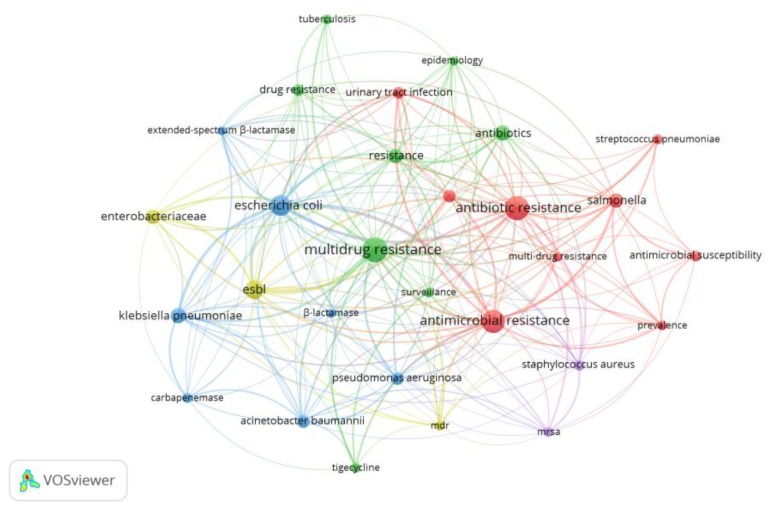
Research topic network visualization of publications related to CA and multidrug resistance in the period between 2008 and 2017. Note: The minimum number of occurrences of a keyword is 20.

In total, 2607 documents were indexed between 1982 and 2017, of which 64.9% (1693 documents) were published in the last 10 years. Figure 5 shows the research topic network visualization of publications related to CA and multidrug resistance in the period between 2008 and 2017. Interestingly, *Acinetobacter baumannii*, *Pseudomonas aeruginosa*, *Enterobacteriaceae*, *Staphylococcus aureus*, *Salmonella* spp. and *Streptococcus pneumoniae* appeared in Figure 5, which are 6 of the 12 bacteria reported by WHO on the global priority list of antibiotic-resistant bacteria [27]. Therefore, researchers related to CA should focus their studies on the previously mentioned bacteria to address and mitigate the harmful effects of the increase in multidrug-resistant bacteria. This is an example of how a bibliometric analysis can be used to suggest changes to policy research or strengthen research relationships between countries and research groups for a common cause. Furthermore, tuberculosis (TB) also appeared in Figure 5. Half a million people are affected by multidrug-resistant TB annually [4,5]. Nevertheless, about 10.4 million fell ill with TB in 2016, and a mortality of 1.4 million people has been reported [28]. Recent reports suggest controlling this deadly pathogen by using β-lactamase inhibitors, such as CA [29].

### 2.4. Quantification of Studies on Clavulanic Acid Production by Streptomyces clavuligerus

In this part of the study, the effect of adding *Streptomyces clavuligerus* to the search was explored since CA is produced by this Gram-positive bacterium. In total, 261 studies related to the production of CA by *S. clavuligerus* were found in the period of 1976–2017. The main subject areas of the indexed documents were (see also Supplementary Materials, Figure S3):

- Biochemistry, Genetics, Molecular Biology.
- Immunology and Microbiology.
- Chemical Engineering.
- Pharmacology, Toxicology, and Pharmaceutics.
- Medicine.

A reorganization of the subject areas was observed when compared to CA studies (Figure 2a and Figure S3). Medicine moved to fifth place—it occupied first place in CA studies.

**Figure 6:**
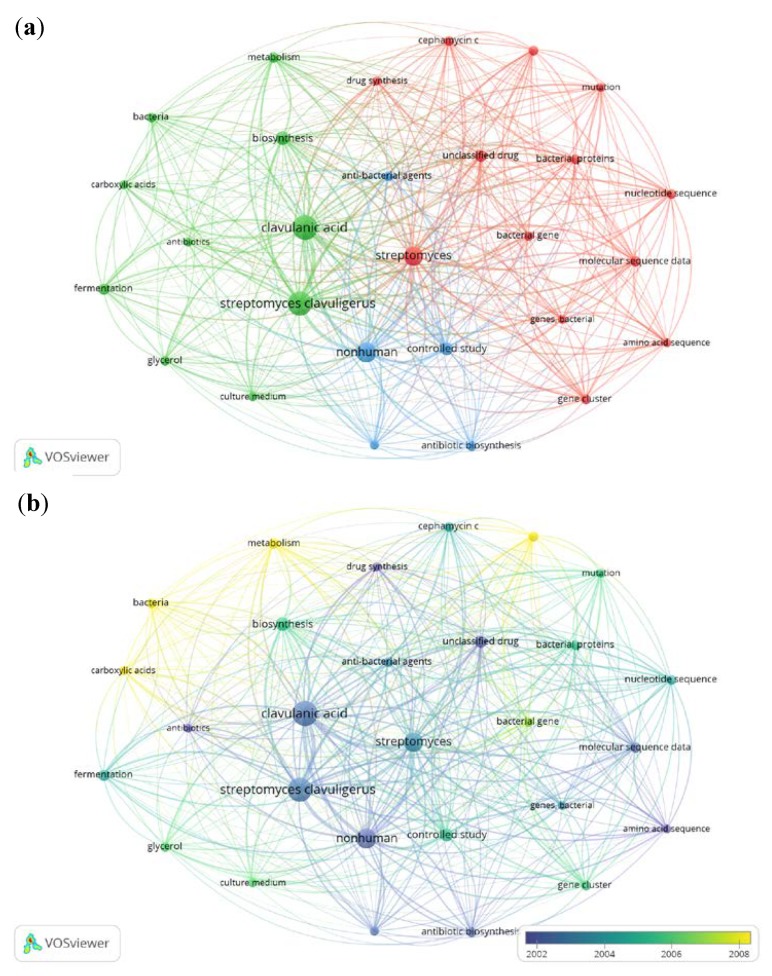
Network of clavulanic acid studies by *Streptomyces clavuligerus* in the period of 1975–2017. (**a**) Research-topic map for publications about clavulanic acid production by *Streptomyces clavuligerus*. (**b**) Research-topic map with time overlay (2002–2008) for studies on clavulanic acid production by *Streptomyces clavuligerus*.

Figure 6a shows the network visualization of CA studies related to *S. clavuligerus* in the period of 1976–2017, in which one can distinguish and catalogue three clusters: (i) molecular biology and bioinformatics (red, 13 items), (ii) metabolism and, design, control and operation of bioprocess (green, 10 items) and (iii) CA biosynthesis and applications (blue, 5 items). These clusters have governed the main CA studies in the last four decades. Nevertheless, Figure 6b shows the topics of studies on CA production by *S. clavuligerus* in the period between 2002 and 2008; at the end of this period, the yellow zone played a preponderant role (this probably due to the intrinsic metabolic complexity of CA synthesis and regulation [8,30,31,32,33]). In fact, genome-scale metabolic models of *S. clavuligerus* have been used to explain the biological complexity of CA synthesis and also to elucidate the genotype–phenotype relationship [32,34,35].

#### 2.4.1. Studies on Chemical Engineering in CA by *S. clavuligerus*

**Figure 7:**
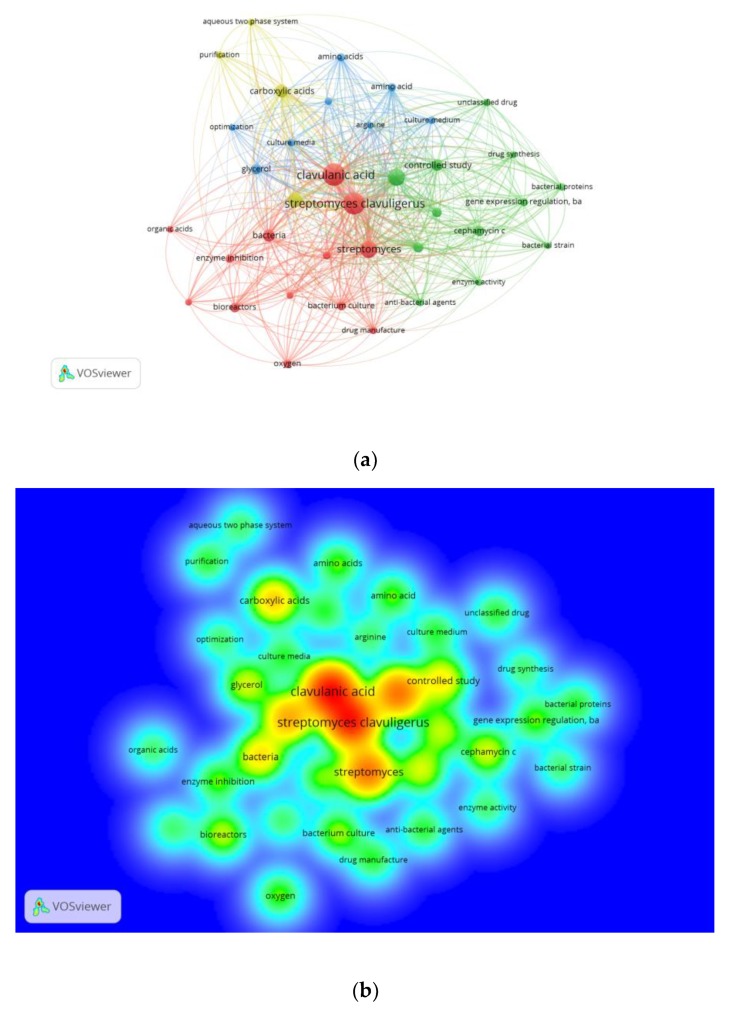
Publications about clavulanic acid production by *Streptomyces clavuligerus* in the chemical engineering discipline between 1976 and 2017. (**a**) Research-topic map for publications about clavulanic acid production by *Streptomyces clavuligerus*. (**b**) Research-topic density map for publications about clavulanic acid production by *Streptomyces clavuligerus*.

Interestingly, chemical engineering appeared in third place, probably due to the strong interconnection it has with other subject areas concerning bioprocess operations, media design, and the optimization process, amongst others. Furthermore, chemical engineering had the highest increase in percentage of publications related to CA production by *S. clavuligerus* (see Table 3). In addition, this is a clear signal of the importance of chemical engineering in CA research in the last 4 decades. Consequently, a bibliometric analysis was conducted for CA studies related to chemical engineering (Figure 7).

Figure 7 shows the research topic network visualization and the research topic density visualization for studies on CA production by *S. clavuligerus* related to the chemical engineering discipline. The research topic network visualization is divided into four clusters which are represented by different colors in Figure 7a. One could catalogue the clusters as:

- Design, control and operation of bioprocess (red, 13 items).
- Molecular biology (green, 12 items).
- Metabolism (blue, 8 items).
- Downstream processing (yellow, 4 items).

Additionally, Figure 7a,b show the study areas of CA production by *S. clavuligerus* on which chemical engineering has been focused in the last 42 years. All of this valuable information provides insights for developing new strategies for creating further collaboration and funding opportunities in the CA field. For instance, in the cluster of molecular biology, the node of gene expression and regulation had a strong interrelation with drug synthesis, CA, cephamycin C and enzyme activity (Figure 7a). In this regard, an engineered CA overproducer *S. clavuligerus* strain was reported with a titer up to 6.69 g/L, becoming the strain with the highest CA titer to date [30,31]. Aside from that, one can observe a relationship between carboxylic acid and CA (Figure 7b). Recently, Ramirez-Malule and coworkers reported a strong association between TCA cycle intermediates accumulation and CA synthesis by *S. clavuligerus*. The authors reported succinate, oxaloacetate, malate and acetate accumulation during CA synthesis in continuous cultivations of *S. clavuligerus* [32].

#### 2.4.2. Leading Authors, Institutions and Countries

**Figure 8:**
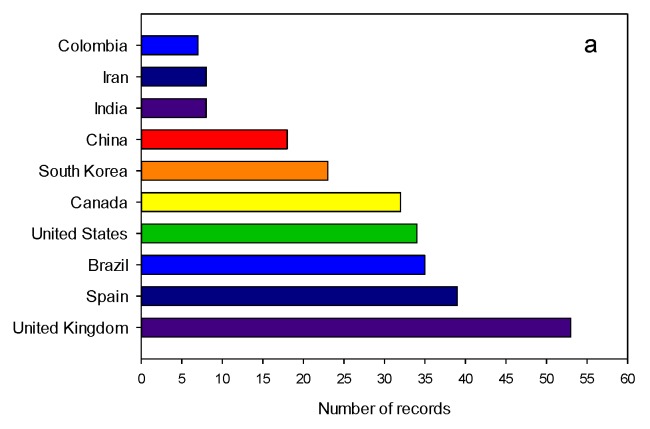

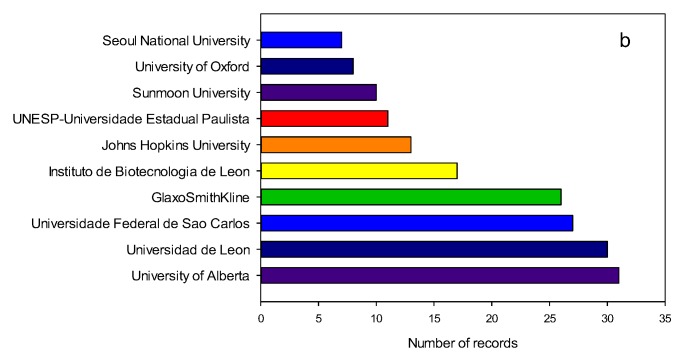
Summary of publications on clavulanic acid production by *Streptomyces clavuligerus* between 1976 and 2017. (**a**) Top 10 leading countries in the field of clavulanic acid production by *Streptomyces clavuligerus*. (**b**) Top 10 leading institutions in the field of clavulanic acid production by *Streptomyces clavuligerus*.

In connection with studies on CA production by *S. clavuligerus*, the United Kingdom, Spain and Brazil are ranked first in terms of research output (Figure 8a). Interestingly, Brazil and Colombia appeared in the top 10 leading countries working on studies on CA production by *S. clavuligerus* with 35 and 7 documents respectively (Figure 8a); this is also in agreement with the top 10 researchers in this field (e.g., Alberto Badino, ranked fifth place) (see Supplementary Materials, Table S7).

Figure 8b shows the ten primary organizations in the field of CA production by *S. clavuligerus*. It is clear that universities play an important role in the academic/scientific production of this field. However, some pharmaceutical companies also contributed to the progress of CA production and research, e.g., GlaxoSmithKline, which commercialized Augmentin®. The University of Alberta is the most productive institution followed by the University of Leon, which is in accordance with the most productive researchers (in this case, Dr. Jensen and Dr. Liras).

**Figure 9:**
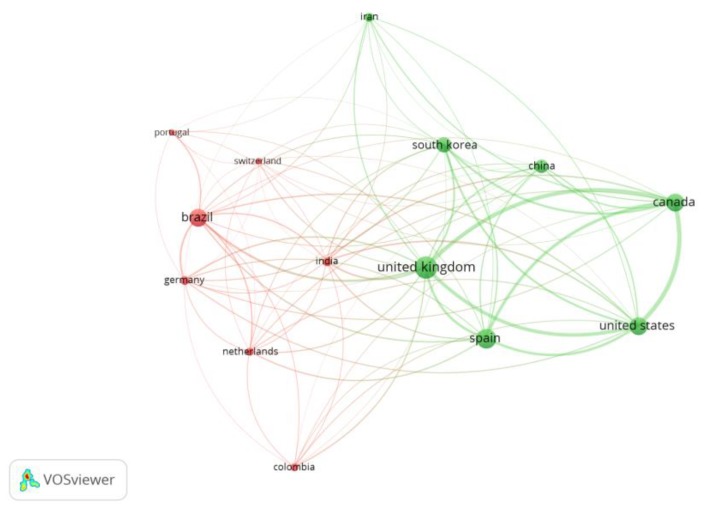
Visualization of the country collaboration network for studies on clavulanic acid production by *Streptomyces clavuligerus*. Note: Countries contributing a minimum number of five documents and citations.

In addition, Figure 9 shows the country collaboration network for studies on CA production by *S. clavuligerus*. Here, two clusters were observed (red and green, see Figure 9); the United Kingdom, the United States, Spain, Canada and Brazil have the strongest collaboration network.

Despite the advances in CA studies and their application in global health, the cost of drugs that contain CA is still high and it is generally not covered by medical insurance, especially in low-income and lower-middle-income countries. This fact, along with the poor living conditions of people worldwide, are responsible for the high rate of deaths caused by infectious disease. This global health problem should encourage the scientific community to develop engineered strains with high CA titer or design new CA-derived antibiotics in order to offer high-quality and cheap drug availability.

## 3. Methods

### 3.1. Database Search and Study Selection

Systematic searches were performed in the Scopus database. The following terms were used in the search:

Study #1

- “Clavulanic acid”. The search was done on 25 April 2018.

Study #2

- “Clavulanic acid” and “clavulanate”. The search was done on 4 September 2018.

Study #3

- “Clavulanic acid” and “Antibiotic resistance”. The search was done on 4 September 2018.

Study #4

- “Clavulanic acid” and “Multidrug resistance”. The search was done on 4 September 2018.

Study #5

- “Clavulanic acid” and “*Streptomyces clavuligerus*”. The search was done on 25 April 2018.

### 3.2. Data Extraction

- The terms were searched in the article title, abstract and keywords.
- Refined by: document types: Article, review, letter, or conference paper.
- Timespan: 1975–2017.
- The related records for each field were recovered and integrated in data files. The information exported from the Scopus database was: (i) citation information, (ii) bibliographical information and (iii) abstract and keywords. The software VOS viewer 1.6.7 was used for data analysis and visualization [36].

## 4. Future Perspectives

A recent study estimated that if antimicrobial drug resistance continues to grow, 10 million people will die annually by 2050, along with a reduction between 2% and 3.5% in gross domestic product. This phenomenon would cost up to 100 trillion USD globally [37]. Clavulanic acid resistance is a global concern. The increase of CA resistance is highly related to the inappropriate use and frequent administration of CA [38,39]. As a future perspective, the design of CA-derived molecules is required to overcome the increase in CA resistance. Thus, genetic engineering applications, transcriptomic and proteomic techniques will lead to advanced process development for the production of CA-derived molecules at an industrial scale. Consequently, strong emphasis on collaboration between companies, research groups and governments must be encouraged towards the development of new CA-derived molecules.

## 5. Conclusions

More than 40,000 documents related to CA have been published in the last 40 years; however, the record of CA publications has been rather constant in the last decade, indicating a stagnation in this field. CA studies showed three trends (1975–1999, 2000–2003 and 2004–2017) based on the publication records. The profile of CA academic studies resembled a sigmoidal profile. The United States, France, the United Kingdom, Spain and Brazil were the leading countries in the scientific production of studies on both CA and CA production by *S. clavuligerus*. The bibliometric analysis of this study allowed the identification of medicine and biochemistry, genetics and molecular biology, as the areas of knowledge with the highest impact on CA and CA production by *S. clavuligerus* studies, respectively. Nevertheless, chemical engineering had the highest increase in percentage of publications related to CA production by *S. clavuligerus*. Antibiotic-resistant bacteria was detected as an emerging area in CA studies—and it has grown rapidly in the last 10 years.

## Figures and Tables

**Table 1:** Top 10 author keywords of CA and clavulanate studies.

Rank	Keywords	Occurrences
1	Antibiotics	101
2	Antibiotic resistance	56
3	Children	54
4	Amoxicillin	41
5	Antimicrobial resistance	38
6	Clavulanic acid	32
7	*Escherichia coli*	31
8	Urinary tract infection	29
9	Antibiotic	29
10	Amoxicillin/clavulanate	27

**Table 2:** Top 10 author keywords of CA and antibiotic resistance studies.

Rank	Keywords	Occurrences
1	Antibiotic resistance	217
2	Antimicrobial resistance	164
3	*Escherichia coli*	134
4	esbl	91
5	Antibiotics	76
6	Resistance	62
7	Urinary tract infection	61
8	*Klebsiella pneumoniae*	60
9	Drug resistance	48
10	Multidrug resistance	46

**Table 3:** Subject areas of studies on CA production by *S. clavuligerus* in the chemical engineering discipline between 1976 and 2017.

Subject Area	1976–1988	1999–2002	2003–2017	Percentage of Variation
Biochemistry, Genetics and Molecular Biology.	43%	70%	64%	49% (↑)
Pharmacology, Toxicology and Pharmaceutics.	40%	10%	14%	−65% (↓)
Medicine.	23%	7%	12%	−48% (↓)
Immunology and Microbiology.	20%	52%	51%	155% (↑)
Chemical Engineering.	6%	10%	27%	350% (↑)

## Supplementary Materials

The following are available online at http://www.mdpi.com/2079-6382/7/4/102/s1. Figure S1: Quantitative growth process of the studies concerning clavulanic acid in the period of 1975–2017 (black line) and its prediction (blue line, sigmoidal equation with three parameters) of studies. Figure S2: Quantitative growth process of the studies concerning amoxicillin-CA resistance between 1978 and 2017 (black line), and its prediction (blue line, exponential growth equation with two parameters) of studies. Figure S3: Documents grouped by areas for clavulanic acid production by *Streptomyces clavuligerus* between 1976 and 2017. Figure S4: Documents grouped by areas for clavulanic acid and clavulanate between 1977 and 2017. Figure S5: Top 10 leading countries in the field of clavulanic acid and clavulanate. Figure S6: Top 10 leading institutions in the field of clavulanic acid and clavulanate. Table S1: Top 10 researchers of CA studies. Table S2: Top-10 author keywords of CA studies in 1987. Table S3: Top 10 author keywords of CA studies in 2002. Table S4: Top-10 author keywords of CA studies in 2010. Table S5: Parameter values of the sigmoidal equation. Table S6: Parameter values of the exponential growth equation. Table S7: Top 10 researchers of CA production by *Streptomyces clavuligerus* studies. Table S8: Top 10 author keywords of CA and antibiotic resistance studies. Table S9: Most cited global research in the field of CA studies by *S. clavuligerus*. Table S10: Top 10 researchers of CA and clavulanate studies.
