# Supplementary material for: Bibliometric Analysis of Global Research on Clavulanic Acid
Source: Antibiotics (Basel). 2018 Nov 26;7(4):102. doi: 10.3390/antibiotics7040102 (PMC6316524; doi:10.3390/antibiotics7040102)
Supplement: Supplementary file 1 [file antibiotics-07-00102-s001.pdf]

Supplementary Material

This Supplementary Material presents some results related to CA studies in the period of time between 1975 and2017, and also studies concerning amoxicillin-CA resistance between 1978 and 2017. Additionally, the mathematical equations that represent the profile of number of publications of both cases in the last four decades are presented.

The top 10 researchers (according to the number of publications) in the field of CA are presented in Table S1. Patrice Nordmann and Ronald Jones are considered the most active researchers in this field, by contributing 213 and 209 documents, respectively.

Table S1. The top 10 researchers of CA studies.

Rank	Authors	Affiliation	No. of publications
1	Nordmann, P.	Swiss National Reference Center for Emerging Antibiotic Resistance (NARA)	213
2	Jones, R.N.	JMI Laboratories Incorporated	209
3	Poirel, L.	Swiss National Reference Center for Emerging Antibiotic Resistance (NARA)	152
4	Brook, I.	Georgetown University	135
5	Jacobs, M.R.	NHS Foundation Trust, Department of Infection	109
6	Livermore, D.M.	Public Health England, Antimicrobial Resistance and Healthcare Associated Infections (AMRHAI) Reference Unit	109
7	Appelbaum, P.C.	Penn State Health Milton S. Hershey Medical Center.	93
8	Labia, R.	Laboratoire Universitaire de Biodiversité et Ecologie Microbienne, Centre de Quimper	93
9	Bonomo, R.A.	Case Western Reserve University	92
10	Baquero, F.	Hospital Ramon y Cajal	83

The top 10 author keywords with their occurrences of CA studies at 1987, 2002 and 2010 are shown in Tables S2, S3, and S4, respectively.

Table S2. The top 10 author keywords of CA studies at 1987.

Rank	Keywords	Occurrences
1	Clavulanic acid	3
2	Ticarcillin	3
3	Timentin	2
4	Pharmacokinetics	2
5	Antibiotic	2
6	Agar disk diffusion test	1
7	Broth dilution test	1
8	Minimal inhibitory concentrations	1
9	*Pseudomonas aeruginosa*	1
10	*Pseudomonas maltophilia*	1

Table S3. The top 10 author keywords of CA studies at 2002.

Rank	Keywords	Occurrences
1	Antibiotics	42
2	Resistance	23
3	Antibiotic resistance	21
4	Pneumonia	16
5	Antimicrobial resistance	16
6	Clavulanic acid	16
7	*Streptococcus pneumoniae*	15
8	Macrolides	14
9	Infection	14
10	Community-acquired Pneumonia	13

Table S4. The top 10 author keywords of CA studies at 2010.

Rank	Keywords	Occurrences
1	Antibiotics	73
2	*Escherichia coli*	44
3	Resistance	38
4	Antimicrobial resistance	35
5	Infection	33
6	Antibiotic resistance	30
7	Children	28
8	*Staphylococcus aureus*	25
9	Pneumonia	25
10	Treatment	22

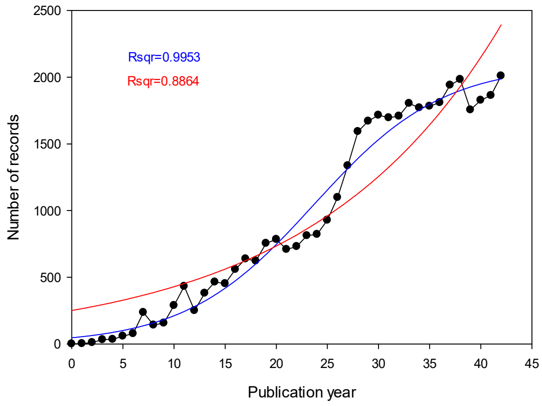

Figure S1. Quantitative growth process of the studies concerning clavulanic acid in the period of 1975-2017 (black line) and its prediction (blue line, sigmoidal equation with three parameters. Red line, exponential growth equation with two parameters) of studies.

The sigmoidal equation with three parameters is the following:

$$y = \frac{a}{1+e^{\left(\frac{-(x-x_0)}{b}\right)}}, \qquad (1)$$

where y is the number of records of publication of CA studies, and x is the publication year. a, b and x_0 are the parameters of the equation. In this study, $Rsqr = 0.9953$.

Table S5. shows the parameter values of the sigmoidal equation and their standard error.

Table S5. Parameters values of the sigmoidal equation.

Coefficient	Value	Standard error
a	2095.5264	80.6085
b	6.2780	0.5006
x_0	23.7193	0.7491

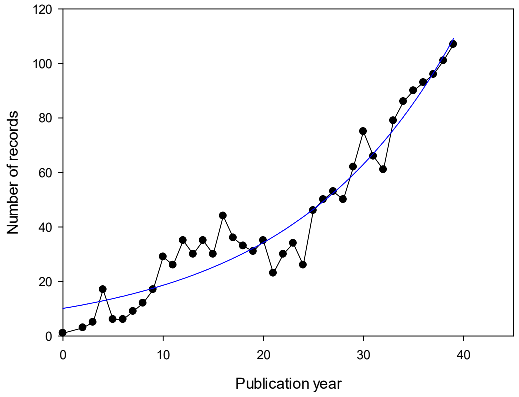

Figure S2. Quantitative growth process of the studies concerning amoxicillin-CA resistance between 1978 and 2017 (black line), and its prediction (blue line, exponential growth equation with two parameters) studies.

The exponential growth equation with two parameters is the following:

$$w = c * e^{(d*t)}, \qquad (2)$$

where w is the numbers of records of publication of amoxicillin-CA resistance studies, and t is the publication year. c and d are the parameters of the equation. In this study, $Rsqr = 0.9317$.

Table S6 shows the parameter values of exponential growth equation and its standard error.

Table S6. Parameter values of the exponential growth equation.

Coefficient	Value	Standard error
C	10.1122	1.1036
D	0.0610	0.0034

The main subject areas of the indexed documents for clavulanic acid production by *Streptomyces clavuligerus* are presented in Figure S3:

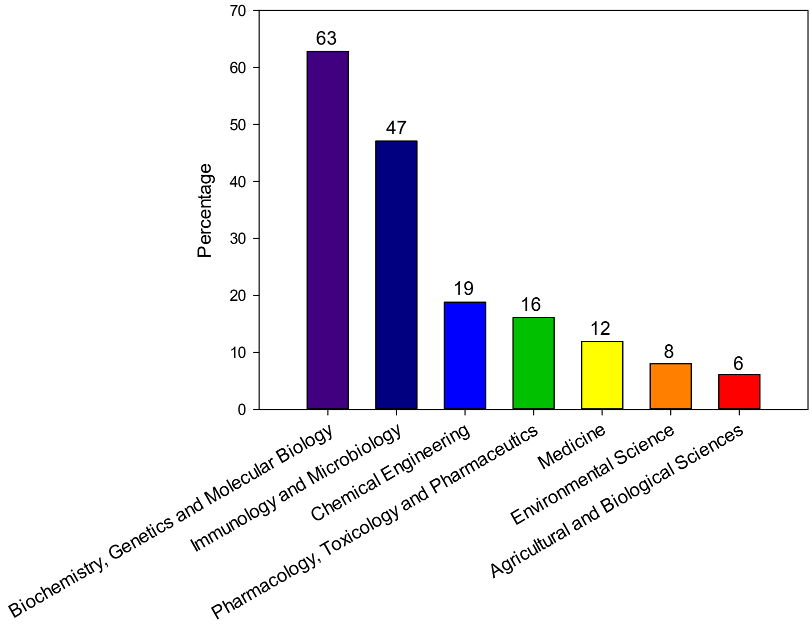

Figure S3. Documents grouped by areas for clavulanic acid production by *Streptomyces clavuligerus* between 1976 and 2017. Note: Percentages add up to more than 100 because a document can be indexed for more than one area. Other areas contributed 14.6%.

The top 10 researchers (in connection with number of publications) in the field of CA production by *Streptomyces clavuligerus* are presented in Table S7.

Table S7. The top 10 researchers of CA production by *Streptomyces clavuligerus* studies.

Rank	Authors	Affiliation	No. of publications
1	Liras, P.	Universidad de Leon, Departamento de Biología Molecular	33
2	Jensen, S.E.	University of Alberta, Department of Biological Sciences	30
3	Hokka, C.O.	Universidade Federal de Sao Carlos, Department of Chemical Engineering	22
4	Martín, J.F	Universidad de Leon, Departamento de Biología Molecular	21
5	Badino, A.C.	Universidade Federal de Sao Carlos, Graduate Program of Chemical Engineering	17
6	Elson, S.W.	GlaxoSmithKline plc, Spain, Parque Tecnológico de Madrid	14
7	Townsend, C.A.	Johns Hopkins University, Department of Chemistry	13
8	Pérez-Redondo, R.	Instituto de Biotecnologia de Leon	11
9	Rodríguez-Gárcia, A.	Instituto de Biotecnologia de Leon,	11
10	Santamarta, I.	Instituto de Biotecnologia de Leon,	11

Table S8. The top 10 author keywords of CA and antibiotic resistance studies.

Rank	Keywords	Occurrences
1	Clavulanic acid	117
2	*Streptomyces clavuligerus*	76
3	*Streptomyces*	10
4	Fermentation	9
5	Glycerol	8
6	β-lactam	7
7	Antibiotic	7
8	*S. clavuligerus*	7
9	Strain improvement	6
10	Regulation	5

Table S9 shows the most cited studies related to CA production by *S. clavuligerus*. Only 5 studies have more than 100 citations.

Table S9. Most cited global research in the field of CA studies by *S. clavuligerus*.

Title	Authors	Publication year	Journal	No. of citation
Clavulanic acid: a beta lactamase inhibiting beta lactam from *Streptomyces clavuligerus* [1]	Reading, C., Cole, M.	1977	Antimicrobial Agents and Chemotherapy 11(5), pp. 852-857	584
Clavulanic acid, a novel β-lactam isolated from *Streptomyces clavuligerus*; X-ray crystal structure analysis [2]	Howarth, T.T., Brown, A.G., King, T.J.	1976	Journal of the Chemical Society, Chemical Communications (7), pp. 266b-267	180
A regulatory gene (ccaR) required for cephamycin and clavulanic acid production in *Streptomyces clavuligerus*: Amplification results in overproduction of both β-lactam compounds [3]	Pérez-Llarena, F.J., Liras, P., Rodríguez-García, A., Martin, J.F.	1997	Journal of Bacteriology 179(6), pp. 2053-2059	129
Purification and Characterization of Clavaminate Synthase from *Streptomyces clavuligerus*: An Unusual Oxidative Enzyme in Natural Product Biosynthesis [4]	Salowe, S.P., Neil Marsh, E., Townsend, C.A.	1990	Biochemistry 29(27), pp. 6499-6508	113
Carbon catabolite regulation of cephalosporin production in *Streptomyces clavuligerus* [5]	Aharonowitz, Y., Demain, A.L.	1978	Antimicrobial Agents and Chemotherapy 14(2), pp. 159-164	112

The main subject areas of the indexed documents for clavulanic acid and clavulanate are presented in Figure S4:

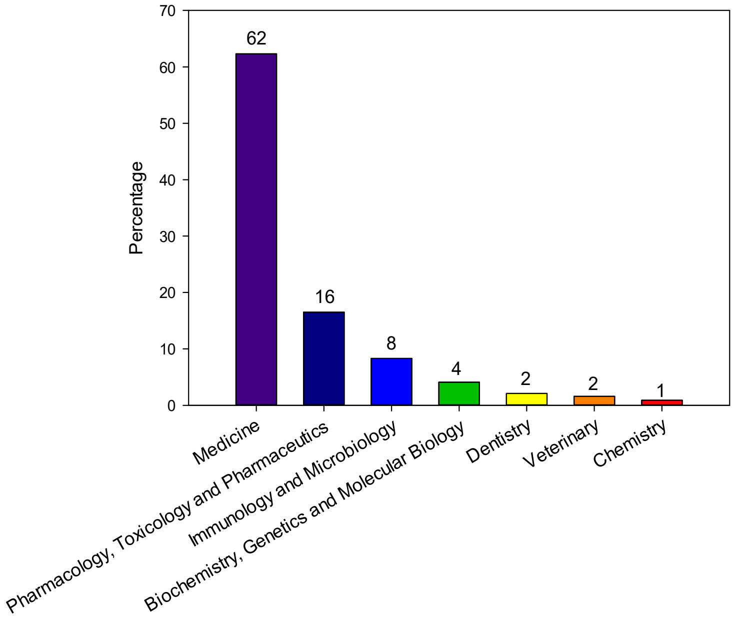

Figure S4. Documents grouped by areas for clavulanic acid and clavulanate between 1977 and 2017. Other areas contributed 4.2%.

Top 10 leading countries in the field of clavulanic acid and clavulanate are presented in Figure S5.

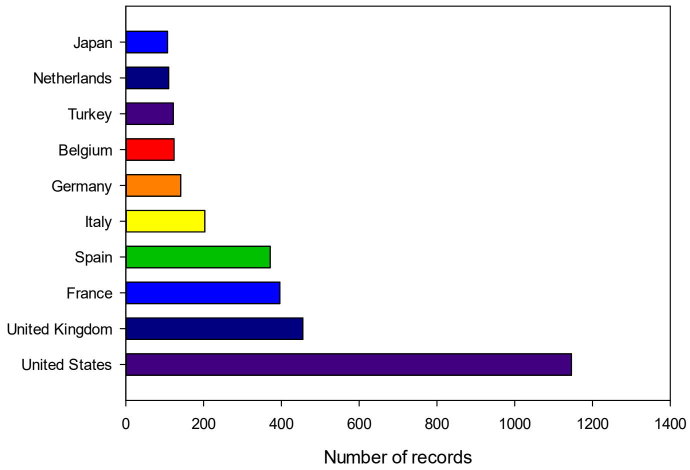

Figure S5. Top 10 leading countries in the field of clavulanic acid and clavulanate.

Top 10 leading institutions in the field of clavulanic acid and clavulanate are presented in Figure S6.

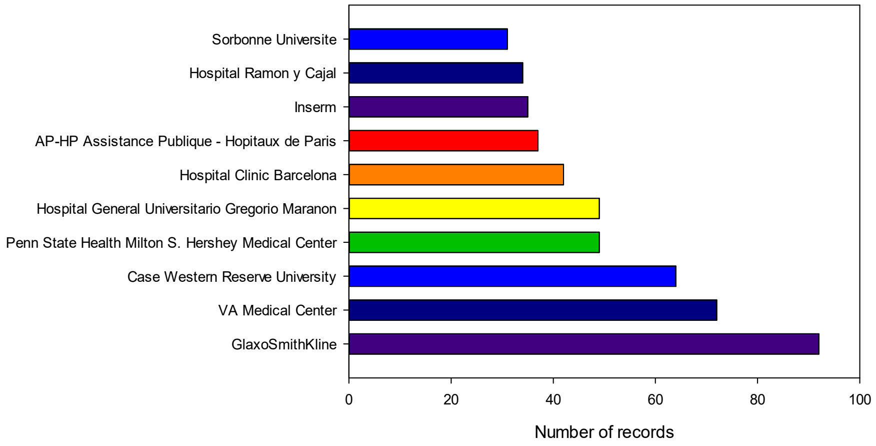

Figure S6. Top-10 leading institutions in the field of clavulanic acid and clavulanate.

The top 10 researchers (according to the number of publications) in the field of CA and clavulanate are presented in Table S10.

Table S10. The top 10 researchers of CA and clavulanate studies.

Rank	Authors	Affiliation	No. of publications
1	Jacobs, M.R.	NHS Foundation Trust, Department of Infection	63
2	Appelbaum, P.C.	Penn State Health Milton S. Hershey Medical Center	52
3	Jones, R.N.	JMI Laboratories Incorporated	32
4	Cohen, R.	Universite Paris-Est	27
5	Pichichero, M.E.	Rochester General Hospital, Center for Infectious Diseases and Immunology	27
6	Livermore, D.M.	Public Health England, Antimicrobial Resistance and Healthcare Associated Infections (AMRHAI) Reference Unit	25
7	Aguilar, L.	PRISM-AG	22
8	Baquero, F.	Hospital Ramon y Cajal	22
9	Thornsberry, C.	Eurofins Medinet	22
10	Bonomo, R.A.	Case Western Reserve University	21

Reference

1. Reading, C.; Cole, M. Clavulanic Acid: a Beta-Lactamase-Inhibiting Beta-Lactam from *Streptomyces clavuligerus*. *Antimicrob. Agents Chemother.* **1977**, *11*, 852–857.
2. Howarth, T. T.; Brown, A. G.; King, T. J. Clavulanic acid, a novel β-lactam isolated from *Streptomyces clavuligerus*; X-ray crystal structure analysis. *J. Chem. Soc., Chem. Commun.* **1976**, 266b–267.
3. Pérez-Llarena, F. J.; Liras, P.; Rodríguez-García, A.; Martín, J. F. A regulatory gene (ccaR) required for cephamycin and clavulanic acid production in *Streptomyces clavuligerus*: amplification results in overproduction of both beta-lactam compounds. *J. Bacteriol.* **1997**, *179*, 2053–2059.
4. Salowe, S. P.; Marsh, E. N.; Townsend, C. A. Purification and characterization of clavaminate synthase from *Streptomyces clavuligerus*: an unusual oxidative enzyme in natural product biosynthesis. *Biochemistry* **1990**, *29*, 6499–6508.
5. Aharonowitz, Y.; Demain, A. L. Carbon catabolite regulation of cephalosporin production in *Streptomyces clavuligerus*. *Antimicrob. Agents Chemother.* **1978**, *14*, 159–164.
